# Intergroup Discrimination as a Predictor of Conflict within the Same Organization. The Role of Organizational Identity

**DOI:** 10.3390/ejihpe10010001

**Published:** 2019-05-28

**Authors:** Samuel Fernández-Salinero, Gabriela Topa

**Affiliations:** 1 Medicine and Surgery, Psychology, Preventive Medicine and Public Health and Medical Microbiology and Immunology Department, Universidad Rey Juan Carlos, 28933 Madrid, Spain; 2 Social and Organizational Psychology Department, The National Distance Education University, 28015 Madrid, Spain

**Keywords:** perceived discrimination, intergroup conflict, organizational identification, group identification

## Abstract

The main purpose of this research is to shed light upon how perception of intergroup discrimination is related to perception of organizational conflict. This phenomenon is mediated by group identification and moderated by organizational identification. The sample was constituted by 466 employees belonging to the staff of Administration and Service of a Spanish public University. Main research results show that perceived discrimination has a direct effect on organizational conflict through group identification. The relationship between perceived discrimination and group identification is moderated by organizational identification.

## 1. Introduction

Great inner complexity characterizes current organizations. These are usually constituted by numerous units or workgroups [1]. This complexity makes certain subjects identify more with their workgroups than with the organization as a whole [2]. It is a known fact that based on the classic minimal group paradigm, intergroup divisions leads to intergroup favouritism and intra-group rivalry. Therefore if total organization is perceived as an exo-group, rivalry phenomena leading to intergroup conflict may appear [3]. Within this framework, perception of discrimination and group identification may be the seed that leads to perception of intergroup conflict.

It is not by chance that in later years forms of organization identification have changed, triggering an identity crisis associated with work. Thus insofar as current precarious conditions discourage identity inversion positive linkage with organization may prove difficult [4]. Two groups of workers which in previous research have shown a degree of Independence and significant differences in their own identities converge within the environment of public university: administration and services staff and teachers-researchers. This research has been based on the ground of Yubero and Morales research [1] in which it was found that administration and services staff show particular identity and cultural organization characteristics.

The main purpose of this research is to shed light upon how perception of intergroup discrimination is related to perception of organizational conflict. This phenomenon is mediated by group identification and moderated by organizational identification within a Spanish public university.

### 1.1. Perceived Discrimination within Organization Environment

Perception of discrimination occurs when a subject notice that is been treated in a different or unfair way according to their group membership [5,6]. It is important to study perceptions of discrimination in workers given that these can affect key factors of human resources and organization development. When subjects perceive that they are treated in an unfair way they can feel alienated, disturbed and show a negative behaviour within their work environment [5,6].

The international labour organization defines discrimination as any distinction, exclusion or preference, carried out based on any race, colour, sex, religion, political opinion, nationality or social status [7]. Discrimination may be carried out direct or indirectly. Following the Wadding and Hendriks directives, indirect discrimination occurs when people involved in the discrimination process don’t have intention to discriminate, but they behave differently depending on the belonging to a specific group. Indirect discrimination has it basis on disadvantages which are created in function of the normative context. This peculiarity may be explained because of the presence of social implicit schemes, such as less valued jobs or kind of activities developed at the workplace.

Benokraitis and Feagin [8] presented a way of understanding discrimination denominated subtle discrimination, which is referred to a tendency to discriminate which typically pass unnoticed [9]. This subtle discrimination may adopt the form of doubts about the employee’s capabilities or isolation [10]. Thus, in the frame of this research, the perception of disrespect or mistreatment may obey to a way of discrimination because of the belonging to a specific group that may pass unnoticed.

Discrimination has been studied within the gender or racial optic. In previous researches it has been linked to hindering job success, job satisfaction, and basic needs fulfilment [11]. However, the employee’s response to discrimination has been less investigated [12,13]. This research starts in this limitation and pretend to contribute to a deeper understanding of the discrimination process.

### 1.2. The Role of Social Identity Theory

Within the framework of social identity theory [14] it is stated that a part of a subject’s self-concept derives from social group membership and that subjects are motivated to achieve a positive social identity. Within the optic of self categorization theory [15] a hierarchical system of self and heterocategorization constituted by different levels of abstraction is posed. For example, a person may be categorized as a member of the organization as a whole or as a member of the workgroup. This categorization can occur simultaneously in a given situation when a person categorize himself into both groups [16]. In consequence, subjects don’t act according to their individual identity instead, their organization conduct is socially placed [17]. Following the social identity theory, some aspects of a subject identity come from the belonging to certain social groups [14]. When a situation stands out the definition of the subject based on the similarity and differences with other groups, it will produce a depersonalization phenomenon. For instance, in an intergroup competitive situation at the workplace, subjects will be motivated to act accord the group beliefs and norms. Turner affirmed that different situations may promote different social identifications and different ways to process information, affectivity and behavior.

The central element of the Social Identity Theory is the individual tendency to achieve a positive self-esteem. This may be accomplished by the maximization of the differences between endogroup and exogroup in those relevant and important characteristics which reflect positively the endogroup [14]. Standing out in those dimensions which are positively rated by the group, a person may gain positive distinctiveness and generate positive social identity [18]. 

Going deeper into this matter, organizational values or attitudes may guide the subject about what is expected and valuable in his workplace or social group, and the individual learns it in the socialization process. The expectative received by the group or society contributes to define the comparative context, shaping the desirable characteristics in a particular context and motivating employees to adequate to this standards. When a subject is not able to reach a positive differentiation, discomfort occurs, which will be analysed in the conflict section.

Belonging to a company with a great number of workers can favour group over organizational identification. This phenomenon is associated to behavioural consequences like perception of intergroup conflict when unfair treatment is perceived. It is for this reason that relationships within the organization must be taken into account, since group identification can lead to intergroup conflict [6,19] and to increase in complaints [20].

In the current study and in accordance with previous researches it is assumed that administration and services staff belonging conducts a clear group identification triggered by hierarchical perception that leads to the definition of specific group skills y specialized roles within the organization. Thus establishing intergroup relationships with another organizational workgroups. Furthermore administration and services staff identity prevails over organizational identity [1].

Following the line of self categorization theory, subjects that activate both identities will allegedly tend to perceive a lower level of intergroup conflict than those strongly identified with the workgroup and weakly with the organization.

### 1.3. Intergroup Conflict as an Organizational Constant

Conflict has an important presence in any context that involves different people, groups or department [3]. Despite that based on current views conflict is perceived as a development opportunity, the destructive side is also present as Deutsch states with his differentiation in constructive and destructive conflicts [21].

Following Thomas [22] definition, conflict is the process which begins when one party perceives that the other has frustrated, or is about to frustrate, some concern of his. On the other hand, Van de Vliert [23] defines conflict as the situation that appears when two individuals, an individual and a group, or two groups, perceive that they are being obstructed or irritated by the other part. He highlights the subjective component of the conflict. Besides, he states that frustration will be attributed to the other individual or group. Conflict may appear unilaterally.

Conflict may lead to some desirable consequences such as raising the activities of the group, the productivity or group cohesion [17]. However, when conflict stagnates, it may lead to some undesirable consequences for the organization and health, such as worsen group performance, satisfaction and group coordination [23]. Due to this, is important to detect conflict as soon as possible, in order manage it and promoting healthier results.

Sherif [24] developed a model which explained the conflict based on the subject´s identification with his group. Based on Sherif’s model, Henry Tajfel proposed that the mere creation of arbitrary categories was a sufficient condition to favor certain effects in the members of the groups such as intragroup favoritism or exogroup homogeneity.

To explain the conflict within the optic of the social identity theory is necessary to establish a we-they categorization that leads to group identity which is the reference point for the comparison. The idea proposed within this framework is that competition and intergroup conflict generates greater cohesion and cooperation within groups due to intragroup standardization, being this phenomenon intensified by conflict since people are motivated to achieve positive social identities.

When inequality is perceived, as in the case of discrimination, subjects can opt to social competition processes through which the endogroup becomes involved in a competitive scenario that can lead to intergroup fight for power. A possibility of resolution lies in the possibility of recategorising the group as a single group of upper hierarchy. In other words if categorized groups have strong organizational identities a decrease in conflict perception will be expected.

Tajfel [14] suggested that if social comparison process produces negative results, the subject would experience dissatisfaction. Negative results refer to discrepancy between the desired status of the group in the important characteristics, and reality. When this happens individuals would activate mechanisms in order to counter this discrepancy. This mechanisms may be cognitive, affective or behavioural. Tajfel distinguish two kind of social comparisons. In the first place, he propose secure comparisons – stable and legitimate – and insecure comparisons – unstable and illegitimate- which may lead to different results over conflict. Stability refers to the perception of possibility to change the current situation. Legitimation refers to the perception that there’s a possible justification to the unfair current situation.

There are two fundamental types of strategies in order to face negative comparisons. In the first place, social mobility strategies are activated when the individual perceives that the social barriers are permeable. Individuals will try to become a member of the group with more positive characteristics such as better salary, working hours, status, etc. Nevertheles, permeability perceptions will tend to inhibit the social mobilization, and stimulate individual coping behaviours as try to leave the current group.

However, when the group change is not possible because of the barriers’ impermeability, the group may activate social change beliefs. Based on this beliefs, people will strive in order to achieve a positive revaluation of the endogroup. We can find two kind of possible reevaluation strategies. In the first place, the group may carry out a redefinition of the comparison values. For example, if salary is a valuable and not changeable attribute, the group may evaluate that they have better working hour conditions. In the other hand, the group may try to change comparative reference group, for example, not comparing with the teachers and researchers group, but comparing themselves with janitors group. 

If reevaluation is not satisfactory, the group can activate competitive strategies in order to achieve a positive social identity and resolve the identity conflict. With this kind of strategies, subjects will be motivated to overpass the reference group in those dimensions positively evaluated. Previous researchers [25] have found that intergroup conflict would be most likely when the status structure is perceived as illegitimate -unequal access to resources-, unstable -situation may change-, and when the group barriers are impermeable -members can’t change their groups-. In the current research, as individuals perceive that they have unequal access to resources and opportunities, because they are viewed as not having the same skills and abilities as upper-status group members, and group mobility is not possible, levels of perceived conflict will raise. Notwithstanding when subjects categorize themselves as member of the same organization, this conflict perception will descend because of sharing identity with upper-status group. In the current research we will try to prove this hypothesis. It is for this reason that in thorough revisions of group conflict previously cited such as Bilbao and Dauder [3] competition prevails in explaining conflict. The authors [3] state the changes of individual positions based on goal achievement to competition positions when entering in conflict stage. In this sense it is also stated that perception of incompatible motivations results in the need to outwit the opponent. All the information contained in this paragraph help us find the most suitable assessment tool in order to understand organizational conflict. 

This perspective allows us to raise the following hypothesis. *Hypothesis 1.* Perception of discrimination will be directly related with perception of intergroup conflict. *Hypothesis 2*: this relationship will be mediated by intergroup identity. *Hypothesis 3*. This mediation will be moderated by organizational identity.

## 2. Materials and Methods 

### 2.1. Sample

The sample was composed by 466 subjects (28.8% of males and 62.0% of women) 11% of the sample chose not to respond to this variable. 20.2% of the sample was between 26 and 35 years of age and 42.1 was between 36 and 45, 31.1% was between 46 and 55 and 4.9% was between 56 and 65 years of age. 1.7% of respondents chose not to respond. Related to organizational factors analysis, 27.5% of the sample has been from 0 to 5 years working in the organization, 18.0% from 6 to 10 years, 10.9% from 11 to 15 years, 26.8% from 16 to 20 years, 10.1% from 21 to 30 years and 5.4% had been more than 30 years in the same organization. 1.3% of the sample chose not to respond. Related to academic training we can find that l 1.1% of the sample had a PhD, 43.6% had a university degree, 15.0% professional training, 27.3% high school diploma, 8.2% secondary school and 2.8% had and undergraduate degree. Furthermore 2.1% of the sample preferred not to answer. Concluding, 69.5% of the sample belonged to administration and services staff as public servants while the 29.2% remaining was temporary hired. 1.3% of the sample decided not to respond.

This study was approved by the committee of ethics in May of 2019. The study participants answered a questionnaire consisting of the scales described in the tool section. The questionnaire was delivered by private letter to the members of the collective along with the corresponding instruction manuals and informed consents. The participants fulfilled the questionnaires voluntarily and deposited them in the designated letterboxes of different campuses. Data was analysed by Statistic Software SPSS v.24 through the regression analysis with the PROCESS macro written by Andrew Hayes [26].

### 2.2. Instruments

The questionnaire was composed of several items from other scales used in the fields of organisational psychology collected in the variables described below.

**Perceived discrimination**. An adaptation of Lipponen, Helkama, Olkkonen y Juslin [27] scale was used. The adaptation of this scale was extracted from Topa and Morales [28]. We kept 3 out of 6 used items for the study achieving alpha = 0.83 reliability. Moreover, previous researches have used this procedure obtaining alpha = 0.70 which is an acceptable value. Instrument consisted in a Likert-type scale from 1 (strongly disagree) to 5 (strongly agree). Examples of items are *“Administrative and services staff are not respected enough within the organization”* or *“In general terms I think that administrative and services staff are treated as second rate staff”*. Note that in order to guarantee confidentiality organization appears in place of the name of the real organization. The same criteria was used with regard to organizational and group identity.

**Intergroup conflict**. Intergroup Competition Scale [29] was used. An adaptation of its items was carried out so as to adjust it to the objectives of the current research as previous researches support where this adaptation showed alpha= 0.87 [28]. In our research, the scale reliability was alpha = 0.73 and the response format was Likert-type from 1 (strongly disagree) to 5 (strongly agree). Examples of these items are: *“I think there are communication problems between administrative and services staff and teachers and researchers”,* or *“Teachers and researchers are constantly remarking how good administration and services´ working conditions are”.*

**Organizational and group identity.** These variables were measured with a slightly modified version of the highly popular Mael and Ashforth scale [29]. The value of this scale is unquestionable being one of the most commonly used to this end. Based on previous studies [29] the scale was adapted to measure organization and group identity. The first scale provided alpha= 0.72 reliability, whereas the latter provided alpha = 0.86. The complete instrument reliability coefficient was alpha= 0.80 which can be considered of Good consistency. The original adaptation used by Topa and Morales provided alpha= 0.74 and alpha= 0.75, respectively, which may be considered as an acceptable index according to George and Mallery affirmations [30]. The response format was liker-type from 1(strongly disagree) to 5 (strongly agree) examples of these items are: *“I feel identified with the organization”*, “*I refer to the organization as us instead of them”* or *“I identify with administrative and services staff”*.

## 3. Results

In order to confirm the hypotheses proposed, a model of moderated mediation was designed following Hayes´s indications [26]. Firstly to clarify the exposition variable correlations will be exposed, thereupon mediation model will be tested in order to confirm moderated mediation hypothesis. Through bootstrapping procedure 1000 samples will be extracted randomly from data with the objet to confirm hypothesis with 95% confidence interval. The following table (Table 1) shows correlations between observable variables.

### 3.1. Mediation Analysis

Firstly, direct effects on perception of discrimination (X) were evaluated over conflict (Y). The significant direct effect of both variables can be observed (B = 0.47, SE = 0.23, 95% CI [0.42; 0.51], *p* < 0.001). Hence, upon attempting to understand how this interaction occurs, the mediation model is tested, evaluating the indirect effect of perceived discrimination (X) over conflict (Y) mediated by group identification (M). The mediation model was significant, being the indirect impact of X on Y positive (B = 0.13, SE = 0.01, 95% CI [0.00; 0.03]). Therefore it can be concluded that mediation occurs and that group identification can lead to group conflict.

### 3.2. Moderation Analysis

Once the moderation has been tested it is interesting to evaluate the second part of our model, that is to say whether group identification (M) in participants is moderated by organizational identity (W) when discrimination is perceived (X). The results of the moderation analysis support this, being the sign of the interaction negative (B = −0.09, SE= 0.03, 95% CI [−0.15; −0.03], *p* < 0.01). So, identify with the organization when discrimination is perceived would be inverse related to group identification.

### 3.3. Mediated Moderation Analysis

**Figure 1:**
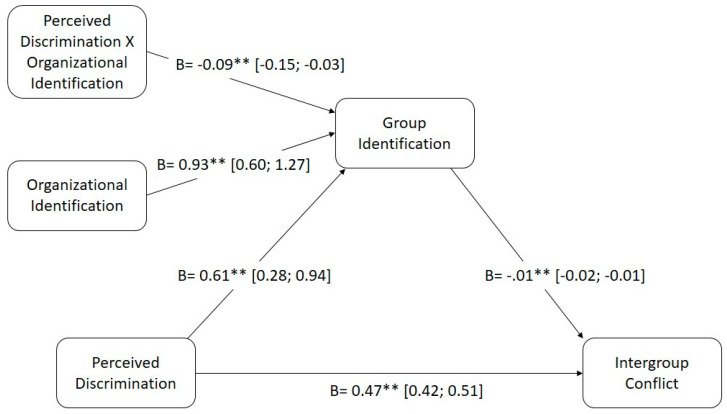
Moderated mediation results. Relationship between perceived discrimination and intergroup conflict through group identification is moderated by organizational identification. (Note: * *p* < 0.05 ** *p* < 0.01.)

Lastly, being the two previous analysis significant, and making the mediated moderation model possible, we can affirm that the model is statistically significant, being the relationship negative. Going deeper into this, perceived discrimination (X) show and effect on intergroup conflict (Y), through group identification (M), being this relationship moderated by organizational identification (W) (B = −0.01, SE = 0.00, 95% CI [−0.02; −0.00]). Therefore, organizational identification contributes to minimize the effects of perceived discrimination over intergroup conflict. Specifically, we can clearly appreciate a lower values in the conflict perception when organizational identification raises (16th percentile 95% CI [0.01; 0.04]; 50th percentile 95% CI [0.01; 0.03], 84th percentile 95% CI [0.00; 0.02]) (Figure 1).

## 4. Discussion

The main purpose of this research is to shed light on how the intergroup perceived discrimination is related to the intergroup conflict, being this phenomenon mediated through group identification and moderated by organizational identification. The current research was carried out in a public Spanish university. This research contributes to increase the understanding of organizational identification, aiding to understand the phenomena which underlies the intergroup conflict in the workplace. 

Discrimination has been studied within the gender or racial optic, but it is important to know that it may happen due to strictly organizational structural factors. In addition, discrimination has been studied related to job satisfaction or organizational commitment [31]. On the other hand, previous researchers [19] have performed approximations related to perceived discrimination effects, but not taking organizational identification into account. Our research shed a new way in understanding the job discrimination. 

Alderfer [32] posed that people insert in groups that are inserted in the wider social system. Our research is based in this premise in order to understand organizational dynamics. 

On the other hand, going deeper into the conflict dynamic, our research shed light about why it may happen inside the same organization. Following the approach of previous investigations [3] there are new discriminative categories that are being added to the classical based on gender and race. Moreover, following Van den Vliert research [23], we confirm that in organizations where the power distance is short, intergroup conflict may appear easily. 

Our research has some limitations. First of all, our sample may not be representative because it has been obtained in only one public university. Due to this we can’t guarantee the ecological validity of the research. It may be interesting to repeat the research in non-university environment.

Another limitation may be the brevity of perceived discrimination measure. Despite the factorial analysis confirms the reliability, we think that a more exhaustive measure may help us to a deeper understanding of the phenomenon we are facing. Moreover, conflict is evaluated through a self-report measure, that doesn’t take into account objective phenomena. This limitation may require the data collection through structured observation, or another types of instruments, which may improve the conflict understanding with greater accuracy.

Future research lines should investigate the impact of perceived discrimination in other variables such as burnout. Recent researches [33] have pointed that perceptions of distributive, procedural, and interpersonal justice have negative indirect effects on turnover intention through burnout and job satisfaction. Besides, distributive, procedural, and interpersonal justice perceptions relate to lower levels of burnout, which in turn promote greater job satisfaction and lower turnover intention among employees. It would be interesting to assess the influence of perceived discrimination over these variables. Future research shall investigate the conflict perception of the privileged group.

On the other hand, as Tufekci [34] pointed, organizational conflict has a significant relation with burnout. Since perceived discrimination leads to conflict, it may be interesting to examine the relationship between this two variables. Besides, it may be interesting to research if there are individual dispositional patterns which may interact with the discrimination evidences. 

Concluding, the current research may help us to guide, construct and manage teams. Different management styles over work teams may lead to significant influences over intergroup conflict. Leaders shall promote a healthy organizational identification, in order to avoid the group team identifications which this research has demonstrated lead to organizational conflict. 

## 5. Conclusions

Our paper demonstrate that perceived discrimination shows a clear influence over the intergroup conflict perception. Specifically, this relationship happens when subjects categorize themselves as a member of a workgroup instead of a member of the organization as a whole. Moreover, when subjects activate the two main categories –organization, workgroup- the intergroup conflict perception fades. As Morales and Yubero [1] shown, group identification conducts to a distance with the other workgroups and to a lack of identification with the organization as a whole. We have demonstrated that group identification takes a very important place in the attitudes towards the other group composed by teachers and researchers.

In addition, following Turner affirmations [35], the self may be categorized in different abstraction levels simultaneously. Multiple identifications are very important inside the organizational models [36]. The current research confirms the affirmations of Onorato y Turner [37] which indicates that the activation of the different identity levels tends to operate in opposition according to its relative importance [10]. Our research confirms the Social Identity approach referred to recategorization as a way of solving the intergroup conflict.

Managers shall be aware of that illegitimate and stable categorizations within the same organization may lead to organizational conflict. On the other hand, this research shows how the presence of group identification, instead of organizational identification, when one group is perceiving discrimination, is related to an increase of perceived conflict. In order to achieve healthier organizations we shall be aware of this phenomena and develop ways to tackle the conflict and build stronger and cohesive workgroups.

## Figures and Tables

**Table 1:** Descriptive statistics and correlations matrix. (N = 466).

Variables	M	SD	α	1	2	3	4
1. Perceived discrimination	4.95	1.43	0.83	-			
2. Intergroup conflict	4.85	1.00	0.73	0.69 **	-		
3. Organizational Identity	5.19	1.15	0.72	−0.11 *	−0.02	-	
4. Group Identity	5.59	1.07	0.86	0.15 **	0.23 **	0.44 **	-

Note: * *p* < 0.05. ** *p* < 0.01.
